# Statin use and the risk of chronic kidney disease in patients with psoriasis: A nationwide cohort study in Taiwan

**DOI:** 10.1371/journal.pone.0237816

**Published:** 2020-08-25

**Authors:** Kwei-Lan Liu, Wen-Chien Tsai, Hung-Pin Tu, Chih-Hung Lee

**Affiliations:** 1 Department of Dermatology, Kaohsiung Chang Gung Memorial Hospital and Chang Gung University College of Medicine, Kaohsiung, Taiwan; 2 Huang PH Dermatology and Aesthetics, Kaohsiung, Taiwan; 3 Department of Public Health and Environmental Medicine, College of Medicine, Kaohsiung Medical University, Kaohsiung, Taiwan; Universita degli Studi Magna Graecia di Catanzaro, ITALY

## Abstract

**Background:**

Psoriasis is associated with hyperlipidemia. Few studies have examined the association among psoriasis, hyperlipidemia, and chronic kidney disease (CKD). It remains a topic of debate whether statin treatment for hyperlipidemia prevents the development of CKD in patients with psoriasis.

**Objective:**

We investigated whether there is an association among psoriasis, hyperlipidemia and CKD. If so, we asked whether statin treatment for hyperlipidemia reduces the risk of CKD in patients with psoriasis.

**Methods:**

A Taiwan nationwide population-based cohort study between 1997 and 2010 included 2,912 patients with psoriasis and 8,736 matched patients without psoriasis (1:3 propensity score matched according to age, sex, and region); 104,609 patients without psoriasis but with hyperlipidemia and 104,609 matched patients without psoriasis or hyperlipidemia (1:1). The hazard ratios, relative risks, and 95% confidence intervals were calculated using Cox proportional hazards model.

**Results:**

Psoriasis significantly increased the risk of CKD (adjusted hazard ratio 2.48, 95% confidence interval 1.81–3.40), and so did hyperlipidemia (adjusted hazard ratio 2.93, 95% confidence interval 2.79–3.08). Compared to treatment without statins, statin treatment for hyperlipidemia reduced the risk of CKD in patients with psoriasis (adjusted relative risk 0.58, 95% confidence interval 0.55–0.62).

**Conclusion:**

As well as hyperlipidemia, psoriasis significantly increased the risk of CKD. Statin treatment for hyperlipidemia reduced the risk of CKD in patients with psoriasis.

## Introduction

Psoriasis, traditionally viewed as a chronic inflammatory disorder of the skin, has far-reaching systemic effects.[1] Although psoriasis was once known as a disease confined to the skin and joints, epidemiologic data have provided compelling evidences of associations between psoriasis and multiple systemic comorbidities, including cardiovascular diseases, metabolic syndrome, gastrointestinal diseases, kidney disease, malignancy, infection, affective disorders, and psoriatic arthritis.[1–3] In these common comorbidities, diabetes and hypertension are the main risk factors for chronic kidney disease (CKD) in the general population.[4,5] Although the existence of a “psoriatic nephropathy” has been proposed on the basis of case reports of glomerulonephritides in patients with psoriasis,[6] the association between psoriasis and kidney disease remains largely unclear. Most studies examining the risk of kidney disease in patients with psoriasis had a small sample size, a cross-sectional design, and varying results.[7]

Dyslipidemia is more prevalent in patients with psoriasis than in patients without psoriasis.[8–10] Dyslipidemia is associated with atherosclerosis, glomerulosclerosis, and tubulointerstitial injury in animal models of CKD.[11] Statins, the 3-hydroxy-3-methyl-glutaryl coenzyme A reductase inhibitor, are widely prescribed for reducing low-density lipoprotein cholesterol and subsequent cardiovascular disease events.[12] A meta-analysis indicated that statin treatment did not reduce the risk of kidney failure events in patients not receiving dialysis but reduced proteinuria and the rate of estimated glomerular filtration decline.[13] However, statin treatment reduced the risk of end-stage renal disease (ESRD) in patients with systemic lupus erythematosus and hyperlipidemia in a nationwide cohort study.[14] Overall, there is no doubt that statin treatment is effective for hyperlipidemia, but current evidence is insufficient for the recommendation of routine statin use for kidney protection.[15] The effect of statins on CKD remains debated. Statins may exert a protective effect on the kidney of a particular patient population. This study investigated whether statin treatment for hyperlipidemia reduces the risk of CKD in patients with psoriasis. We hypothesized that statin treatment for hyperlipidemia is associated with a reduced risk of CKD in patients with psoriasis. To test the hypothesis, we compared the risks of CKD and ESRD between patients with psoriasis receiving statins for hyperlipidemia and those receiving treatment without statins.

## Methods

### Data source and study population

The study project was reviewed and approved by the Institutional Review Committee of Chang Gung Memorial Hospital in Taiwan. We conducted a retrospective cohort study by using data from the National Health Insurance Research Database (NHIRD) for the period between 1^st^ January 1997 and 31^st^ December 2010. The National Health Insurance program provides care for approximately 99% of the Taiwanese population over 23 million people. For this analysis, we used data from Longitudinal Health Insurance Database (LHID) of 2010, which includes the claims data of one million individuals randomly selected from all insurants in the NHIRD. In the NHIRD, diagnosis coding was performed according to the International Classification of Diseases, Ninth Revision, Clinical Modification (ICD-9-CM) diagnostic criteria. The information was managed using a double-scrambling protocol, through which the original identification number of each patient was encrypted to protect privacy while maintaining consistency. All data was de-identified, and researchers analyzed data anonymously under the ambit of personal data protection laws.

### Inclusion and exclusion criteria (Fig 1)

In total, 2,912 patients with psoriasis were identified, and 8,736 matched patients without psoriasis (at a ratio of 1:3) were included in the psoriasis cohort; 104,609 patients without psoriasis but with hyperlipidemia and 104,609 matched patients without psoriasis or hyperlipidemia (at a ratio of 1:1) were included in the hyperlipidemia cohort. The inclusion criteria were patients newly diagnosed with psoriasis, without psoriasis but with hyperlipidemia, and age ≥ 20 years. The study population was followed up for 14 years. The exclusion criteria were age < 20, psoriasis or hyperlipidemia diagnosed before 1997, psoriasis or hyperlipidemia diagnosed in 2010 (< 1-year follow-up), psoriasis of clinic visits < 3, hyperlipidemia of clinic visits < 3, hyperlipidemia diagnosed before psoriasis, CKD diagnosed before hyperlipidemia, CKD diagnosed before psoriasis, and CKD diagnosed before 1997.

**Fig 1:**
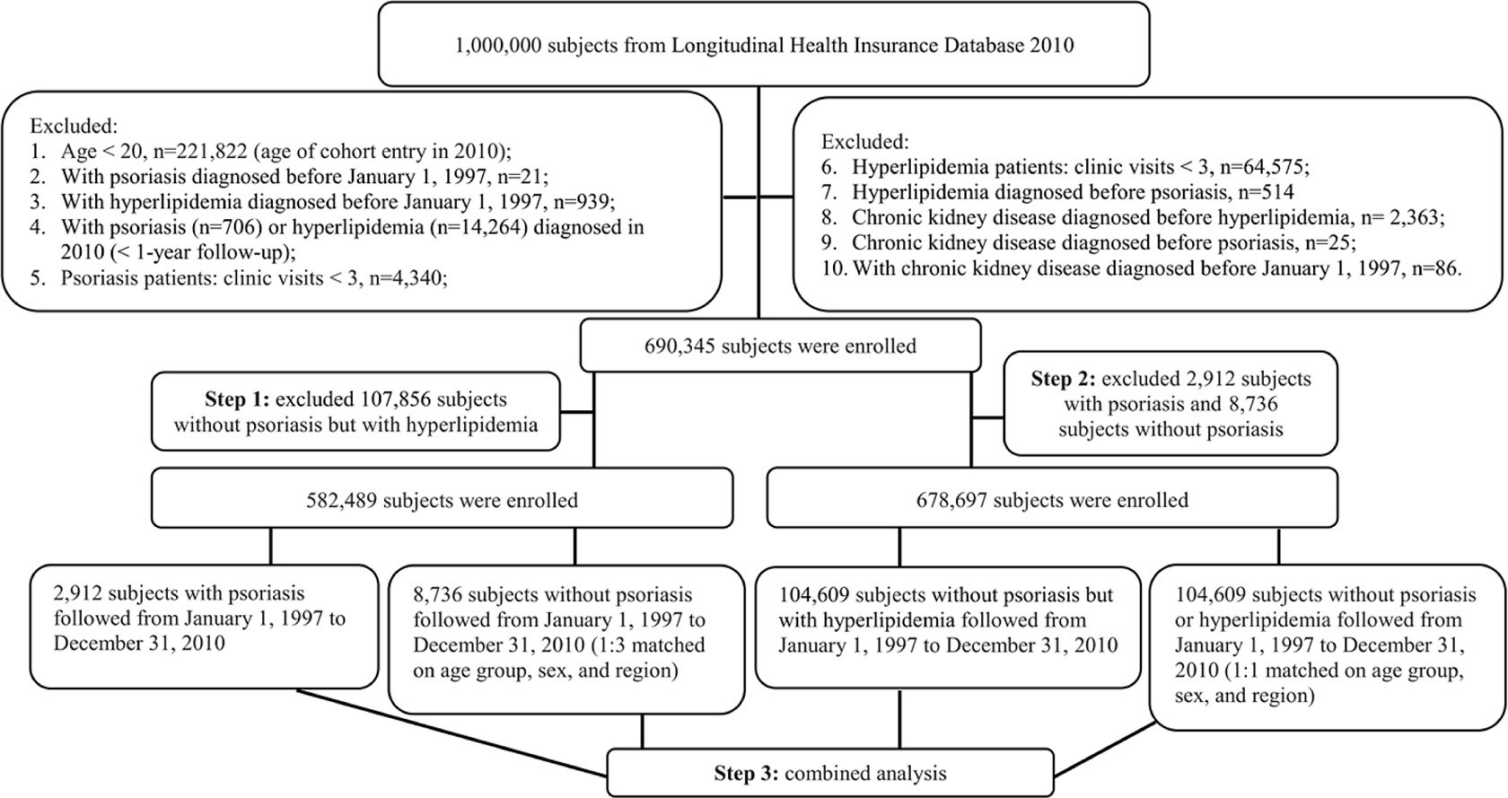
Flow chart presenting study patients.

### Ascertainment of psoriasis and hyperlipidemia

The definitions of psoriasis and hyperlipidemia were made by a physician-recorded primary diagnosis (ICD-9-CM 696.1 and 696.0; ICD-9-CM 272) at an outpatient or inpatient visit. The diagnosis of psoriasis or hyperlipidemia was defined as ≥ 3 clinic visits.

### Outcome assessment of CKD and ESRD events

CKD event was defined as the new occurrence of CKD (ICD-9-CM 585.x and 586.x) at an outpatient or inpatient visit. Patients with CKD ≥ 3 clinic visits were favored to have the accurate ICD-9-CM diagnosis of CKD. In patients with CKD, Taiwan’s Catastrophic Illness Certificate is delivered to those who need long-term dialysis treatment, which represents a definite diagnosis of ESRD. To make sure the accurate diagnosis in capturing ESRD, ESRD events were defined as the new appearance of patients with a Taiwan’s Catastrophic Illness Certificate for ESRD at an outpatient or inpatient visit.

### Statin medications

The doses of statin were converted to the number of defined daily doses (DDDs) as defined by the World Health Organization.[16] Statin users were defined by receiving statins more than 28 cumulative defined daily dose (DDD). The DDD recommended by the World Health Organization is a method of standardizing drug dose across multiple drug types so that they can be compared. Cumulative DDD (cDDD) was estimated as the sum of the dispensed DDD of any statin. Average statin dose, or DDD per day, was calculated as cDDD divided by total days of drug prescription. Simvastatin 20 mg, lovastatin 20 mg, pravastatin 20 mg, fluvastatin 80 mg, atorvastatin 10 mg, or rosuvastatin 10 mg was calculated as 1 DDD (S1 Table).

### Comorbidities

In addition to the demographic risk factors of age, sex, and region, we evaluated potential confounding factors, including hypertension, myocardial infarction, congestive heart failure, peripheral vascular diseases, cerebrovascular diseases, dementia, chronic pulmonary disease, rheumatic diseases, peptic ulcer disease, mild liver disease, diabetes without chronic complication, diabetes with chronic complication, hemiplegia or paraplegia, any malignancy, including leukemia and lymphoma, except malignant neoplasm of skin, moderate or severe liver disease, metastatic solid tumor, and acquired immune deficiency syndrome/human immunodeficiency virus infection.

### Statistical analysis

Continuous and categorical variables were analyzed using the t-test and chi-squared test, respectively. Comparisons of statin use between patients with psoriasis and patients without psoriasis but with hyperlipidemia were calculated using Wilcoxon rank sum test. The incidence rate ratio (IRR) was calculated using the PROC GENMOD generalized linear model to perform Poisson regression analysis, which is a log-linear model. The matching variables of age, sex, and region were used in the STRATA statement to allow each unique variable to define a stratum. The Kaplan-Meier method was used to estimate the survival curves for each group, and the log-rank test was used to test for homogeneity among the survival curves. Propensity score matching was used to incorporated potential risk factors, including age, sex, and region into the model. The hazard ratios (HRs), adjusted relative risks (RRs), and 95% confidence intervals (CIs) were calculated using Cox proportional hazards model. The variables with a significant level of *P* < 0.05 were entered to this model. All statistical analyses were performed using the SAS statistical software (version 9.4, SAS Institute, Cary, NC, USA). All statistical tests were two-sided with a significance level of *P* < 0.05.

## Results

### Characteristics of the study population

A total 2,912 patients with psoriasis and 8,736 matched patients without psoriasis were included in the psoriasis cohort (Fig 1). The characteristics of study and matched patients in each cohort were shown in Table 1. The age, sex, and region of all study and matched patients were similar. The mean age ± standard deviation was 49.0 ± 16.7 years in patients with psoriasis. In the 2,912 patients with psoriasis, there were 455 patients (15.6%) with hyperlipidemia, 276 (60.7%) under statin treatment, 71 (2.4%) with CKD, and 10 (0.3%) with ESRD. The median follow-up duration was 7.8 years with an interquartile range 4.9–10.6 years. Hypertension (26.1%), peptic ulcer disease (16.1%), mild liver disease (12.7%), and chronic pulmonary disease (12.5%) were common comorbidities in patients with psoriasis.

**Table 1:** Characteristics of study and matched patients.

	Psoriasis cohort			Hyperlipidemia cohort		
	Patients with psoriasis	Matched patients	*P* value	Patients without psoriasis but with hyperlipidemia	Matched patients	*P* value
N	2912	8736		104609	104609	
CKD, n (%)	71 (2.4)	143 (1.6)	< 0.01**	7096 (6.8)	3928 (3.8)	< 0.01**
ESRD, n (%)	10 (0.3)	29 (0.3)	0.93	1222 (1.2)	704 (0.7)	< 0.01**
Median follow-up duration (IQR), years	7.8 (4.9–10.6)	14.0 (14.0–14.0)	< 0.01**			
Age of cohort entry in 2010, mean (SD), years	49.0 (16.7)	49.0 (17.2)	0.89	60.3 (13.4)	60.3 (13.4)	0.92
Age group, n (%)			0.66			0.99
20 to 30	380 (13.0)	1233 (14.1)		1340 (1.3)	(1.3)	
> 30 to 40	626 (21.5)	1785 (20.4)		5509 (5.3)	5509 (5.3)	
> 40 to 50	595 (20.4)	1785 (20.4)		15731 (15.0)	15731 (15.0)	
> 50 to 60	584 (20.1)	1744 (20.0)		31100 (29.7)	31100 (29.7)	
> 60 to 70	339 (11.6)	996 (11.4)		25436 (24.3)	25387 (24.3)	
> 70	388 (13.3)	1193 (13.7)		25493 (24.4)	25542 (24.4)	
Sex, males, n (%)	1799 (61.8)	5343 (61.2)	0.55	52622 (50.3)	52883 (50.6)	0.25
Region, n (%)			0.21			0.37
Northern	1347 (46.3)	3997 (45.8)		50797 (48.6)	49016 (46.9)	
Central	607 (20.8)	1979 (22.7)		22404 (21.4)	25267 (24.2)	
Southern	844 (29.0)	2435 (27.9)		27769 (26.5)	27009 (25.8)	
Eastern, offshore islets, and others	114 (3.9)	325 (3.7)		3639 (3.5)	3317 (3.2)	
Comorbidities, n (%)						
Hypertension	761 (26.1)	1457 (16.7)	< 0.01**	66982 (64.0)	30029 (28.7)	< 0.01**
Myocardial infarction	9 (0.3)	17 (0.2)	0.26	1307 (1.2)	276 (0.3)	< 0.01**
Congestive heart failure	79 (2.7)	124 (1.4)	< 0.01**	6026 (5.8)	2898 (2.8)	< 0.01**
Peripheral vascular disease	45 (1.5)	98 (1.1)	0.07	4725 (4.5)	1853 (1.8)	< 0.01**
Cerebrovascular disease	138 (4.7)	325 (3.7)	0.01**	11867 (11.3)	5637 (5.4)	< 0.01**
Dementia	22 (0.8)	54 (0.6)	0.43	1199 (1.1)	983 (0.9)	< 0.01**
Chronic pulmonary disease	365 (12.5)	853 (9.8)	< 0.01**	20340 (19.4)	13266 (12.7)	< 0.01**
Rheumatologic disease	96 (3.3)	82 (0.9)	< 0.01**	3324 (3.2)	2061 (2.0)	< 0.01**
Peptic ulcer disease	468 (16.1)	1054 (12.1)	< 0.01**	28150 (26.9)	17242 (16.5)	< 0.01**
Mild liver disease	370 (12.7)	593 (6.8)	< 0.01**	27438 (26.2)	8925 (8.5)	< 0.01**
Diabetes without chronic complication	228 (7.8)	252 (2.9)	< 0.01**	27291 (26.1)	5217 (5.0)	< 0.01**
Diabetes with chronic complication	60 (2.1)	59 (0.7)	< 0.01**	8419 (8.0)	1277 (1.2)	< 0.01**
Hemiplegia or paraplegia	25 (0.9)	57 (0.7)	0.25	1738 (1.7)	1142 (1.1)	< 0.01**
Any malignancy, including leukemia and lymphoma, except malignant neoplasm of skin	112 (3.8)	223 (2.6)	< 0.01**	4978 (4.8)	4370 (4.2)	< 0.01**
Moderate or severe liver disease	7 (0.2)	9 (0.1)	0.08	133 (0.1)	146 (0.1)	0.44
Metastatic solid tumor	9 (0.3)	6 (0.1)	< 0.01**	208 (0.2)	247 (0.2)	0.06
AIDS/HIV	7 (0.2)	8 (0.1)	0.05	86 (0.1)	41 (0.0)	< 0.01**

** Indicates statistical significance at the 0.01 level (2-tailed)

† Comorbidities were defined as ≥ 3 outpatient claims. Data of continuous and categorical variables were analyzed using *t*-test or Wilcoxon rank sum test, and chi-square test or Fisher's Exact test.

‡ CKD: chronic kidney disease; ESRD: end-stage renal disease; IQR: interquartile range; SD: standard deviation; AIDS/HIV: acquired immune deficiency syndrome/human immunodeficiency virus.

### CKD events in the psoriasis cohort

The CKD events per 1,000 person-years were 3.22 in patients with psoriasis (Table 2). Compared to matched patients, the adjusted HR of CKD was 2.48 (95% CI, 1.81–3.40) in patients with psoriasis. A similar result was observed in the sensitivity analysis by 5-year follow-up. The adjusted HR of CKD was 2.46 (95% CI, 1.51–4.01) in patients with psoriasis in the sensitivity analysis by 5-year follow-up.

**Table 2:** Chronic kidney disease events in the psoriasis cohort and the hyperlipidemia cohort.

	CKD events/total patients (%)	Person-years	Events per 1000 person-years (95% CI)	IRR (95% CI)	Adjusted HR (95% CI)	*P* value	β, *P* for interaction†
**Psoriasis cohort**							
Matched patients	143/8736 (1.64)	121508	1.18 (1.17–1.18)	1.00	1.00		
Patients with psoriasis	71/2912 (2.44)	22050	3.22 (3.18–3.26)	2.74 (2.06–3.64)	2.48 (1.81–3.40)	< 0.01	–0.58, 0.12
**Sensitivity analysis (5-year follow-up)**							
Matched patients	37/8736 (0.42)	43608	0.85 (0.84–0.86)	1.00	1.00		
Patients with psoriasis	32/2912 (1.10)	13091	2.44 (2.40–2.49)	2.90 (1.80–4.65)	2.46** (1.51–4.01)	< 0.01	–0.19, 0.10
**Hyperlipidemia cohort**							
Matched patients	3319/104609 (3.17)	1444946	2.30 (2.29–2.30)	1.00	1.00		
Patients without psoriasis but with hyperlipidemia	7096/104609 (6.78)	740387	9.58 (9.56–9.61)	4.17 (4.00–4.35)	2.93** (2.79–3.08)	< 0.01	–0.13, < 0.01
***Men***							
Matched patients	1753/52883 (3.31)	730493	2.40 (2.39–2.41)	1.00	1.00		
Patients without psoriasis but with hyperlipidemia	3963/52622 (7.53)	364359	10.84 (10.84–10.91)	4.53 (4.28–4.79)	3.21** (3.00–3.44)	< 0.01	
***Women***							
Matched patients	1566/51726 (3.03)	714453	2.19 (2.19–2.20)	1.00	1.00		
Patients without psoriasis but with hyperlipidemia	3133/51987 (6.03)	376028	8.33 (8.31–8.36)	3.80 (3.58–4.04)	2.62** (2.44–2.82)	< 0.01	
**Sensitivity analysis (5-year follow-up)**							
Matched patients	965/104609 (0.92)	521098	1.85 (1.85–1.86)	1.00	1.00		
Patients without psoriasis but with hyperlipidemia	4128/104609 (3.95)	453671	9.10 (9.07–9.13)	4.83 (4.50–5.18)	3.37** (3.12–3.63)	< 0.01	–0.31, < 0.01
***Men***							
Matched patients	463/52883 (0.88)	263496	1.76 (1.75–1.76)	1.00	1.00		
Patients without psoriasis but with hyperlipidemia	2304/52622 (4.38)	226277	10.18 (10.14–10.22)	5.68 (5.14–6.28)	3.93** (3.53–4.37)	< 0.01	
***Women***							
Matched patients	502/51726 (0.97)	257601	1.95 (1.94–1.96)	1.00	1.00		
Patients without psoriasis but with hyperlipidemia	1824/51987 (3.51)	227394	8.02 (7.99–8.05)	4.04 (3.66–4.46)	2.84** (2.56–3.16)	< 0.01	

** Indicates statistical significance at the 0.01 level (2-tailed)

† Cox proportional hazards regression model was applied after adjustment for covariates.

‡ IRR was calculated by using PROC GENMOD to perform Poisson regression analysis.

§ HRs with 95% CIs and their *P* values were calculated after adjustment for comorbidities in Table 1 by using Cox proportional hazards regression model. Age, sex, and region were used in the STRATA statement such that each unique value for age, sex, and region defines a stratum.

¶ CKD: chronic kidney disease; IRR: incidence rate ratio; HR: hazard ratio; CI: confidence interval.

**Fig 2:**
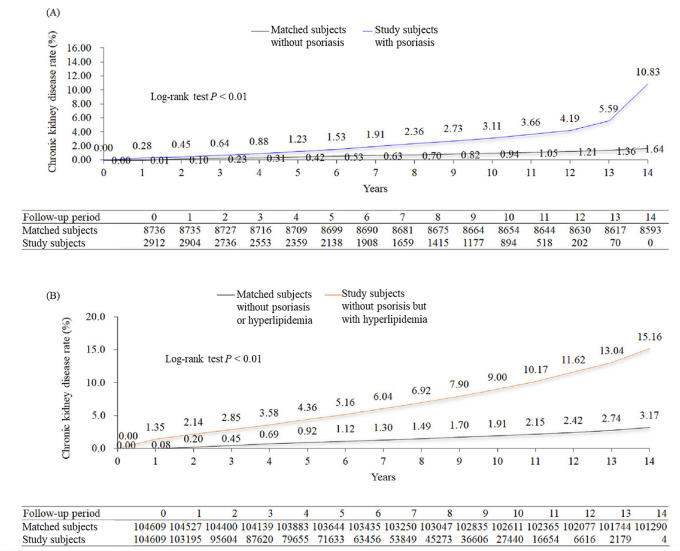
Cumulative incidences of chronic kidney disease in (A) the psoriasis cohort and (B) the hyperlipidemia cohort. Differences in the incidences of chronic kidney disease were compared using the log-rank test and Kaplan-Meier analysis.

During the follow-up period, CKD was significantly increased in patients with psoriasis. By 14-year follow-up period, the cumulative incidences of CKD were 10.83% in patients with psoriasis and 1.64% in matched patients (*P* < 0.01) (Fig 2A).

### CKD events in the hyperlipidemia cohort

The CKD events per 1,000 person-years were 9.58 in patients without psoriasis but with hyperlipidemia (Table 2). Compared to matched patients, the adjusted HR of CKD was 2.93 (95% CI, 2.79–3.08) in patients without psoriasis but with hyperlipidemia. Similar results were observed in the sensitivity analysis by 5-year follow-up. The adjusted HR of CKD was 3.37 (95% CI, 3.12–3.63) in patients without psoriasis but with hyperlipidemia in the sensitivity analysis by 5-year follow-up. A gender-specific relationship of CKD risk was observed. Compared to matched patients, the adjusted HRs of CKD were 3.93 (95% CI, 3.52–4.37) for male and 2.84 (95% CI, 2.56–3.16) for female patients without psoriasis but with hyperlipidemia.

CKD events significantly increased in patients without psoriasis but with hyperlipidemia during the follow-up period. By 14-year follow-up period, the cumulative incidences of CKD were 15.6% in patients without psoriasis but with hyperlipidemia and 3.17% in matched patients (*P* < 0.01) (Fig 2B).

### Statin treatment for hyperlipidemia and the risk of CKD in patients with psoriasis

Compared to treatment without statins, statin treatment for hyperlipidemia reduced the risk of incident CKD. Similar results were observed in the analyses of CKD ≥ 3 clinic visits and CKD ≥ 1 clinic visits (Table 3). In the analysis of CKD ≥ 3 clinic visits, patients receiving statin had a significantly lower risk of CKD than patients receiving treatment without statins. The adjusted RR was 0.50 (95% CI, 0.26–0.96) in patients with psoriasis receiving statins.

**Table 3:** Statin treatment for hyperlipidemia and the risk of incident chronic kidney disease.

	CKD events (%)	Total patients	Adjusted RR (95% CI) †	*P* value
**CKD ≥ 1 clinic visits**				
Hyperlipidemia cohort receiving treatment without statins	3477 (8.2)	42163	1.00	
Hyperlipidemia cohort receiving statins	3619 (5.8)	62446	0.52** (0.49–0.54)	< 0.01
Psoriasis cohort receiving treatment without statins	11 (6.1)	179	0.63 (0.35–1.13)	0.12
Psoriasis cohort receiving statins	16 (5.8)	276	0.48** (0.29–0.78)	< 0.01
**CKD ≥3 clinic visits**‡				
Hyperlipidemia cohort receiving treatment without statins	1726 (4.3)	40412	1.00	
Hyperlipidemia cohort receiving statins	2202 (3.6)	61029	0.58** (0.55–0.62)	< 0.01
Psoriasis cohort receiving treatment without statins	8 (4.5)	176	0.84 (0.42–1.69)	0.63
Psoriasis cohort receiving statins	9 (3.3)	269	0.50* (0.26–0.96)	0.04

* Indicates statistical significance at the 0.05 level (2-tailed)

** Indicates statistical significance at the 0.01 level (2-tailed)

† The adjusted RR was compared to hyperlipidemia cohort receiving treatment without statins.

‡ CKD < 3 clinic visits was excluded.

§ Crude RR with 95% CIs and their *P* value was calculated by using Cox proportional hazards regression model. The adjusted RR and 95% CI were estimated after adjustment for covariates in Table 1 by using a stepwise Cox proportional hazards regression method. The variables with a significance level of *P* < 0.05 were entered to this model.

¶ CKD: chronic kidney disease; RR: relative risk; CI: confidence interval

The association between statin use and the risk of incident ESRD in patients with CKD was neither consistent nor significant in most strata because of the small sample size of ESRD events in each stratum. Compared to patients receiving treatment without statins, statin treatment reduced the risk of incident CKD but no dose-dependent protective effect was found (Table 4). In patients without psoriasis, the adjusted RRs of CKD were 0.39 (95% CI, 0.32–0.47), 0.38 (95% CI, 0.35–0.41), 0.49 (95% CI, 0.45–0.54), and 0.69 (95% CI, 0.66–0.74) in the average statin doses of 0<DDD<28, 28≤DDD≤168, 168<DDD≤336, and DDD>336 groups, respectively. In patients with psoriasis, the adjusted RR of CKD was 0.25 (95% CI, 0.08–0.78) in the average statin dose of 28≤DDD≤168 group. The cutoff of 28 DDD was determined by the prescription routine of 4 weeks (4 x 7 days) in the healthcare system in Taiwan. The cutoff of 168 DDD was determined by the cumulative medical use of 6 months (6 x 28 days). The cutoff of 336 DDD was determined by the cumulative medical use of 12 months (12 x 28 days).

**Table 4:** Statin treatment for hyperlipidemia and the risk of incident CKD without dose-dependent protective effect.

	CKD events (%)	Total patients	Adjusted RR (95% CI)	*P* value
**Hyperlipidemia receiving treatment without statins**	3488 (8.2)	42342	1.00	
**Hyperlipidemia receiving statins**				
Average statin dose in patients without psoriasis				
0<DDD<28	100 (3.5)	2853	0.39 (0.32–0.47)**	< 0.01
28≤DDD≤168	916 (3.5)	26121	0.38 (0.35–0.41)**	< 0.01
168<DDD≤336	644 (5.3)	12047	0.49 (0.45–0.54)**	< 0.01
DDD>336	1959 (9.1)	21425	0.69 (0.66–0.74)**	< 0.01
Average statin dose in patients with psoriasis				
0<DDD<28	0 (0.0)	16	NA	NA
28≤DDD≤168	3 (2.5)	119	0.25 (0.08–0.78)*	0.02
168<DDD≤336	5 (9.1)	55	0.72 (0.30–1.73)	0.46
DDD>336	8 (9.3)	86	0.69 (0.34–1.38)	0.29

* Indicates statistical significance at the 0.05 level (2-tailed)

** Indicates statistical significance at the 0.01 level (2-tailed)

† Cumulative DDD was estimated as the sum of the dispensed DDD of any statin. Average statin dose, or DDD per day, was calculated as cDDD divided by total days of drug prescription. Simvastatin 20 mg, lovastatin 20 mg, pravastatin 20 mg, fluvastatin 80 mg, atorvastatin 10 mg, or rosuvastatin 10 mg will be calculated as 1 DDD (S1 Table).

‡ The adjusted RR and 95% CI were estimated after adjustment for covariates in Table 1 by using a stepwise Cox proportional hazards regression method. The variables with a significance level of *P* < 0.05 were entered to this model.

§ CKD: chronic kidney disease; RR: relative risk; CI: confidence interval; DDD: defined daily dose; NA: not applicable.

## Discussion

In this nationwide study, we observed that patients with psoriasis had a significantly increased risk of CKD during the 14-year follow-up, supporting the existence of “psoriatic nephropathy.” Statin treatment for hyperlipidemia was associated with a reduced risk of CKD in patients with psoriasis.

Our study showed that patients with psoriasis had a higher risk of CKD than matched patients (Table 2). Kidney can be affected by psoriasis through three different mechanisms, including immune-mediated, drug-related, and chronic renal damage.[17] The risk of CKD increasing with the severity of psoriasis may suggest an immune-mediated mechanism. Patients with severe psoriasis may have a higher inflammatory burden.[18] Each large cohort study in the United Kingdom and Taiwan demonstrated that psoriasis was associated with an increased risk of CKD development.[19,20] The cohort study in the United Kingdom found that moderate-to-severe psoriasis was associated with an increased risk of moderate-to-advanced CKD and ESRD, independent of traditional risk factors such as age, sex, body mass index, cardiovascular diseases, diabetes, hypertension, hyperlipidemia, and nephrotoxic medications.[19] In the study, the adjusted HRs of incident CKD were 1.05, 0.99, and 1.93 in the cohorts of overall, mild, and severe psoriasis, respectively.[19] The cohort study in Taiwan similarly found high severity of psoriasis increased the risks of glomerulonephritis, CKD, and ESRD.[20] Psoriasis was an independent risk factor for CKD in the cohorts of all psoriasis (HR 1.28–1.51), mild psoriasis (HR 1.30–1.52), and severe psoriasis (HR 1.23–1.55).[20] Drug-related renal damage is another possible mechanism of CKD in patients with psoriasis. In our study, we did not exclude patients with psoriasis receiving nephrotoxic drugs, such as methotrexate and cyclosporine, which are commonly accepted as the definition of severe psoriasis. The cohort study in the United Kingdom found the association between moderate-to-severe psoriasis and moderate-to-advanced CKD and ESRD was independent of nephrotoxic medications.[19] Concomitant medications and the risks of CKD in patients with psoriasis were investigated in the above-mentioned Taiwanese cohort study.[20] The study investigated acitretin, methotrexate, cyclosporine, azathioprine, and nonsteroidal anti-inflammatory drugs (NSAIDs), among which NSAIDs had the strongest association with CKD in patients with psoriasis.[20] However, NSAIDs were over-the-counter drugs and easily-accessible in Taiwan. The real condition of NSAID use was difficult to evaluate in studies using LHID. As for chronic renal damage, the increased incidence of glomerulonephritis in patients with psoriasis may partly contribute to an increased risk of CKD.[20] We found that the cumulative incidences of CKD in patients with psoriasis significantly increased with the follow-up period (Fig 2). Evidence has shown that the clinical relevance of the absolute risk of CKD attributable to psoriasis increased with age.[19]

We demonstrated that patients with hyperlipidemia had an increased risk of CKD, while patients receiving statin treatment had a reduced risk of CKD (Table 3). Because hyperlipidemia and statin treatment had opposite effects on the risk of CKD, the results were less confounded by indication bias in this study. Apart from an intrinsic cholesterol-lowering effect, statins also exhibit anti-inflammatory, antioxidant, immunomodulatory, endothelial-protective, and plaque-stabilizing capacities that act in concert to prevent from cardiovascular damage.[21] Statins may have potential benefit for renal function regardless of the effect on lowering cholesterol levels.[21] However, the effect of statins on CKD progression remains a topic of debate. Two meta-analyses demonstrated similar results that statins reduced proteinuria but did not significantly reduce the risk of kidney failure events.[13,22] Similar to our study, statin treatment had no protective effect on ESRD or decline of renal function in patients with CKD.[23]

Statin treatment for hyperlipidemia reduced the risk of incident CKD but no dose-dependent protective effect was found (Table 4). One of the possible explanations is the small sample size after stratification according to the cDDD of statins. There were few studies focusing on the dose effect of statins in patients with psoriasis. In a post-hoc analysis, high-dose atorvastatin significantly reduced cardiovascular events compared to standard or low-dose statins in patients with psoriasis.[24] In a meta-analysis, high-dose statins were found to improve the rate of estimated glomerular filtration decline in patients with CKD not requiring dialysis, but standard and low-dose statins were not.[25] Further studies are needed to investigate the role of high-dose statins for CKD prevention in patients with psoriasis.

Compared to previous small and cross-sectional studies, our study has the strength of a large sample size and a longer follow-up period. Several limitations in the present study merit discussion. First, the severity of psoriasis by body surface area and the stages of CKD by actual lab values were not available in the database. Although we did not stratify the severity of psoriasis and the stages of CKD for analysis, our study provided a generalized epidemiological association between unspecified severity of psoriasis and unspecified stages of CKD. Also, the diagnoses of hyperlipidemia and CKD were made by the diagnostic codes and medication records due to lack of laboratory data in the database. The diagnosis relied on administrative claims data reported by physicians. Second, overall medication adherence was unknown. We presumed that all medications were taken as prescribed although some degree of non-compliance was expected. The effectiveness of the therapy in lowering serum lipid levels could not be assessed. Third, we did not consider lifestyle factors, including body mass index, diet, alcohol, smoking, and exercise, because these data were not available in the NHIRD. Previous study indicated that these factors contributed far less to CKD than traditional risk factors, such as older age, diabetes, and hypertension,[26] which had been included in our analysis. Finally, the study was conducted in Taiwanese patients. As well as the risk of CKD in patients with psoriasis, the effect of statins on the risk of CKD may vary with countries and ethnicities.

## Conclusions

We observed an association between statin treatment for hyperlipidemia and a reduced risk of CKD in patients with psoriasis. A formal randomized controlled trial is indicated to clarify the effect of statin treatment on the process of CKD. For patients with psoriasis and hyperlipidemia, our study may serve as a basis for further studies to investigate the effect of statin use on the kidney.

## Supporting information

S1 Table Details of statin treatment for hyperlipidemia in the psoriasis cohort and the hyperlipidemia cohort. (DOCX)
