# Supplementary material for: Statin use and the risk of chronic kidney disease in patients with psoriasis: A nationwide cohort study in Taiwan
Source: PLoS One. 2020 Aug 25;15(8):e0237816. doi: 10.1371/journal.pone.0237816 (PMC7447019; doi:10.1371/journal.pone.0237816)
Supplement: S1 Table — (DOCX) [file pone.0237816.s001.docx]

**S1 Table.** **Details of statin treatment for hyperlipidemia in the psoriasis cohort and the hyperlipidemia cohort**

|  | Psoriasis cohort, n=2921 | Hyperlipidemia cohort, n=104609 | *P* value |
| --- | --- | --- | --- |
| Statin use, n (%) | 276 (60.7) | 62446 (59.7) |  |
| Age (years), median (IQR) | 60.9 (53.9–72.0) | 61.4 (54.0–71.2) | 0.80 |
| C10AA01: Simvastatin, n | 92 | 23991 |  |
| Total clinic visits (frequencies), median (IQR) | 5.0 (2.0–9.5) | 4.0 (2.0–9.0) | 0.62 |
| Total drug use (days), median (IQR) | 130.0 (56.0–238.0) | 112.0 (56.0–240.0) | 0.54 |
| Total dosage, median (IQR), mg | 2170.0 (900.0–4340.0) | 1900.0 (840.0–4480.0) | 0.75 |
| Total dosage/total dosage use days | 20.0 (16.3–20.0) | 20.0 (16.6–20.0) | 0.69 |
| C10AA02: Lovastatin, n | 71 | 19592 |  |
| Total clinic visits (frequencies), median (IQR) | 3.0 (2.0–8.0) | 3.0 (2.0–8.0) | 0.72 |
| Total drug use (days), median (IQR) | 90.0 (30.0–207.0) | 90.0 (30.0–210.0) | 0.95 |
| Total dosage, median (IQR), mg | 1680.0 (600.0–4140.0) | 1800.0 (600.0–4200.0) | 0.84 |
| Total dosage/total dosage use days | 20.0 (20.0–20.0) | 20.0 (20.0–20.0) | 0.28 |
| C10AA03: Pravastatin, n | 42 | 11539 |  |
| Total clinic visits (frequencies), median (IQR) | 4.0 (2.0–11.0) | 4.0 (2.0–8.0) | 0.27 |
| Total drug use (days), median (IQR) | 94.5 (42.0–280.0) | 90.0 (42.0–210.0) | 0.41 |
| Total dosage, median (IQR), mg | 1770.0 (840.0–4880.0) | 1480.0 (600.0–3460.0) | 0.17 |
| Total dosage/total dosage use days | 19.8 (10.0–20.0) | 17.9 (10.0–20.0) | 0.32 |
| C10AA04: Fluvastatin, n | 47 | 11848 |  |
| Total clinic visits (frequencies), median (IQR) | 3.0 (1.0–9.0) | 4.0 (2.0–9.0) | 0.15 |
| Total drug use (days), median (IQR) | 84.0 (30.0–240.0) | 112.0 (56.0–252.0) | 0.15 |
| Total dosage, median (IQR), mg | 4480.0 (2240.0–13440.0) | 6720.0 (2400.0–15040.0) | 0.25 |
| Total dosage/total dosage use days | 75.0 (40.0–80.0) | 72.7 (40.0–80.0) | 0.64 |
| C10AA05: Atorvastatin , n | 137 | 31172 |  |
| Total clinic visits (frequencies), median (IQR) | 5.0 (3.0–11.0) | 5.0 (2.0–12.0) | 0.67 |
| Total drug use (days), median (IQR) | 125.0 (60.0–308.0) | 140.0 (56.0–336.0) | 0.59 |
| Total dosage, median (IQR), mg | 1210.0 (580.0–3230.0) | 1430.0 (580.0–3530.0) | 0.46 |
| Total dosage/total dosage use days | 10.0 (8.6–10.0) | 10.0 (9.2–10.0) | 0.32 |
| C10AA07: Rosuvastatin, n | 72 | 16684 |  |
| Total clinic visits (frequencies), median (IQR) | 4.0 (2.0–12.0) | 5.0 (2.0–10.0) | 0.91 |
| Total drug use (days), median (IQR) | 115.0 (56.0–336.0) | 133.0 (56.0–280.0) | 0.90 |
| Total dosage, median (IQR), mg | 705.0 (290.0–2500.0) | 980.0 (420.0–2050.0) | 0.47 |
| Total dosage/total dosage use days | 6.7 (5.0–10.0) | 9.1 (5.1–10.0) | 0.02 |

† *P* values were calculated by using Wilcoxon rank sum test to compare the data of patients with and without psoriasis.

‡ IQR: interquartile range.
